# Gastroenterologists Reveal More Digestive Symptoms in COVID-19 Patients than Nongastroenterologists in Fever Clinic

**DOI:** 10.1155/2020/8853922

**Published:** 2020-11-30

**Authors:** Yudong Jiang, Chaoqun Han, Tao Bai, Shengyan Zhang, Jun Song, Xiaohua Hou

**Affiliations:** Division of Gastroenterology, Union Hospital, Tongji Medical College, Huazhong University of Science and Technology, 1277 Jiefang Avenue, Wuhan, Hubei Province, China 430022

## Abstract

The incidence of digestive symptoms may vary depending on doctors' professional backgrounds when they inquired suspected COVID-19 patients in a fever clinic. We sought to understand the characteristics of inquiries about digestive symptoms by doctors in different specialties; therefore, inquiry records of 2 gastroenterologists and 6 nongastroenterologists were reviewed. We compared the difference in inquiry of digestive symptoms (diarrhea, vomit, distension, anorexia, and abdominal pain) between these two groups among identified COVID-19 patients. And we further compared the difference of digestive symptoms between confirmed patients and suspected cases who excluded from COVID-19. Among 495 confirmed COVID-19 cases (254 cases by gastroenterologists and 241 cases by nongastroenterologists), 22.83% patients experienced various digestive symptoms in the gastroenterologists' group, while only 4.47% reported digestive symptoms by nongastroenterologists (*p* < 0.0001). Additionally, among initially suspected 611 patients who presented with similar respiratory symptoms inquired by gastroenterologists, confirmed cases presented far more frequency of digestive symptoms than excluded cases (22.8% vs. 3.64%, *p* < 0.0001). Furthermore, confirmed patients reported more percentage of watery diarrhea (56% vs. 36%, *p* < 0.0001) and higher frequent vomit (2.77 ± 0.97 vs. 1.80 ± 0.45 per day, *p* = 0.041) than excluded cases. We concluded that gastroenterologists could detect a greater proportion of gastrointestinal symptoms in COVID-19 patients during fever clinic inquiries. Moreover, confirmed COVID-19 patients are more likely to have higher severity in digestive symptoms than excluded cases. Therefore, physicians in fever clinic should pay more attention to the triage of gastrointestinal symptoms.

## 1. Introduction

The Coronavirus disease 2019 (COVID-19) has spread worldwide now and has been declared an international public health emergency by the World Health Organization (WHO) [1]. Up to now, the infection has caused more than 12 million infections and over 60 thousand deaths. Therefore, early screening and quarantine for COVID-19 patients has become a vital procedure to prevent further spread [2]. The typical clinical manifestations gained wide concerns for viral pneumonia and respiratory manifestations [3, 4]. However, recent evidences indicated that the infection could also present with only or accompanied with digestive symptoms such as diarrhea, vomit, nausea, etc. [5–7].

Nevertheless, few studies have been conducted on the digestive symptoms, and the incidence varies significantly among different study populations [6, 7]. Our previous research showed that COVID-19 patients who had combined digestive symptoms shared longer hospital days and delayed recovery [8]. More importantly, viral nucleic acid could also be detected in the feces and 23.29% of patients had positive stools after negative respiratory samples, indicating that “cured” patients may have the possibility of fecal-oral transmission of the virus [7]. The digestive involvement of 2019-nCoV should be recognized.

Fever clinic was instituted by hospitals to manage patients with fever. During the COVID-19 epidemic, doctors from all majors joined the fever clinic to screen and treat suspected COVID-19 patients. They have diagnosed or excluded hundreds of cases a day and made great contribution to disease control. However, the incidence of digestive symptoms may vary depending on doctors' professional backgrounds; some atypical patients may be overlooked and thus may be misdiagnosed [8].

In this study, we reviewed inquiry records of 2 gastroenterologists and 6 nongastroenterologists in a fever clinic. Through their inquiry features of suspected COVID-19 patients' digestive symptoms, we sought to understand the characteristics of inquiries about digestive symptoms by doctors in different specialties and uncover the truth about the incidence of digestive tract diseases on COVID-19.

## 2. Methods

### 2.1. Study Design

This retrospective study was performed at a fever clinic of Union Hospital, Tongji Medical College in Wuhan, China, which was a designated medical institution to manage patients with COVID-19. The study is aimed at analyzing characteristics of inquiries about digestive symptoms by doctors in different specialties and then compare the different percentages of digestive symptoms in diagnosed and excluded patients who presented with similar respiratory symptoms.

This study was approved by the Medical Ethical Review Committee, Union Hospital of Tongji Medical College, Huazhong University of Science and Technology, China ((2020) no. 0033). Written informed consent was waived due to the rapid emergence of this infectious disease, and their information had been anonymized and deidentified.

### 2.2. Inclusion Criteria for Doctors and Patients

We first reviewed inquiry records of 2 gastroenterologists from our department. We then matched each of the two gastroenterologists with a control doctor. The control doctor needs to meet the following criteria: (1) attending doctor, (2) MD degree, (3) clinical experience of more than 3 years, and (4) different majors. Matching was based on sequential doctor's work identification numbers, such that the next outpatient doctor who met the study criteria was enrolled as a control. Finally, 6 physicians from different majors (cardiology, respiratory, nephrology, hematology, endocrinology, and infectious) were included.

**Figure 1:**
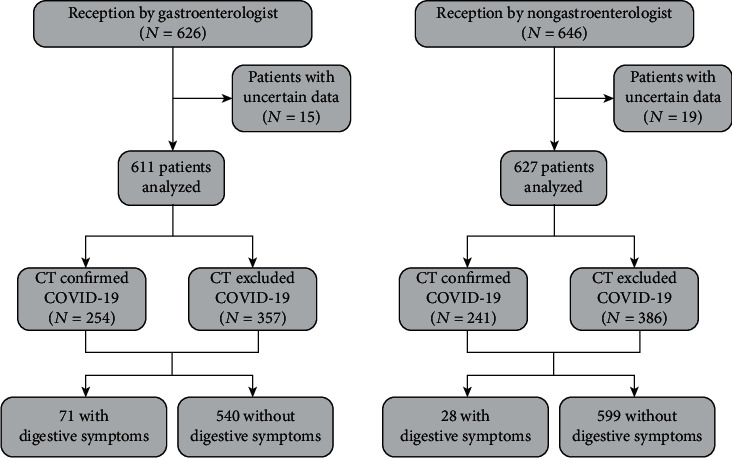
Study protocol and disposition of study patients.

We then searched medical records of 1272 patients (by these eight doctors) who visited between January 02^nd^ and February 23^th^, 2020, the early stage of the domestic pandemic. All definitions are consistent with the “Diagnosis and Treatment Scheme of New Coronavirus Infected Pneumonia” (trial version 6) released by the Chinese National Health Commission and the World Health Organization interim guidance for COVID-19 [9, 10]. The COVID-19 was diagnosed as the imaging findings of viral pneumonia by radiologic CT examinations: bilateral/multifocal or peripheral round ground-glass shadows, superposition with interlobular septal thickening, and paving stone signs [11]. Differential diagnosis was made by detection of influenza A&B and respiratory syncytial virus. Incomplete/uncertain data was excluded. Finally, 495 patients fulfilled the diagnostic criteria of COVID-19 and 743 suspected cases were used for analysis. The protocol of this study is seen in Figure 1.

### 2.3. Data Collection

The demographic and clinical data mainly including presenting clinical symptoms, comorbidity, medical records, laboratory findings, chest CT scans, and viral nucleic acid test for all COVID-19 patients were extracted from electronic medical records. In particular, digestive symptoms (diarrhea, abdominal pain, anorexia, vomiting, distention, heart burn, etc.) were collected in detail. The collected data was reviewed and checked by 3 trained researchers and attending clinicians independently.

### 2.4. Statistical Analysis

All continuous variables were presented as mean ± standard deviation (SD). Categorical continuous variables were presented as numbers and percentages. Two paired *t*-tests were made for continuous variables, and the Chi-square test was applied for categorical variables and percentages. Statistical analysis was performed using SPSS 22 Statistics (IBM Corp, Armonk, NY, USA). A significance level of *p* ≤ 0.05 was used for all models (two-sided). All graphs were drawn by GraphPad Prism 6.0.

## 3. Results

### 3.1. Baseline Characteristics

As showed in Table 1, 611 patients were inquired by gastroenterologists and 627 patients were inquired by nongastroenterologists, respectively. Nearly 40% of patients in the fever clinic were diagnosed with COVID-19. All patients were residents of Wuhan with an average age of 42.24 years. No difference of age and gender was observed, nor any difference of temperature or duration before outpatient visited (*p* > 0.05). Hypertension (4.42%), cardiovascular disease (2.13%), and diabetes (1.96%) were the most common comorbidity in gastroenterologist's inquiries which were of similar proportion to the nongastroenterologists (*p* > 0.05). The proportion of initial symptoms (fever, cough, chest pain, shortness of breath, fatigue, muscle soreness, and diarrhea) were also similar between these two groups (*p* > 0.05).

### 3.2. Gastroenterologists Inquired a Greater Proportion of Digestive Symptoms in COVID-19 Patients

Among 495 confirmed COVID-19 cases (254 by gastroenterologists and 241 by nongastroenterologists), vomit (7.07%), diarrhea (5.86%), and distention (3.03%) were the most common digestive symptoms. As showed in Table 2, there were significantly different percentages of digestive symptoms between these two groups. 58 (22.83%) patients experienced various digestive symptoms in the gastroenterologists' group, while only 18 (4.47%) reported digestive symptoms by nongastroenterologists (*p* < 0.0001). Of them, gastroenterologists revealed significantly higher frequency of distention than nongastroenterologists (4.72% vs. 1.24%, *p* = 0.02). Moreover, significantly higher proportion was observed in diarrhea during inquiry by gastroenterologists (9.84% vs. 1.66%, *p* = 0.0001) although no considerable difference was found in prevalence of abdominal pain, vomit, heartburn, anorexia, and hematochezia (*p* > 0.05). These results suggested that gastroenterologists were more likely to discover higher percentages of digestive symptoms than the nongastroenterologists in COVID-19 patients.

### 3.3. Diagnosed COVID-19 Patients Presented Worse Digestive Symptoms

**Figure 2:**
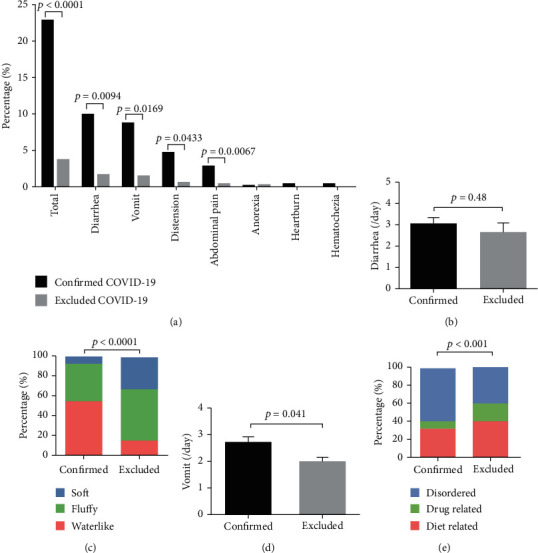
Comparison of digestive symptoms between confirmed COVID-19 and excluded cases. (a) Percentage of digestive symptoms were compared between confirmed COVID-19 cases and excluded cases. (b) No difference was found in stool frequency. (c) Among those who presented with diarrhea, more confirmed patients described watery diarrhea than excluded cases. (d) Confirmed COVID-19 patients had higher frequent vomit than the excluded cases. (e) Higher rates of nonfood or drug-related percentage of disordered vomit were found.

We further compared digestive characteristics between diagnosed and excluded patients among 611 initially suspected COVID-19 who presented with similar respiratory symptoms inquired by gastroenterologists. Interestingly, confirmed COVID-19 cases were far more likely to present digestive symptoms than the excluded ones (58 vs. 13, 22.8% vs. 3.64%, *p* < 0.0001, Figure 2(a)). Significant higher rates of diarrhea (9.84% vs. 1.68%, *p* = 0.0094), vomit (8.66% vs. 1.40%, *p* = 0.0169), distension (4.72% vs. 0.56%, *p* = 0.0433), and abdominal pain (2.76% vs. 0.28%, *p* = 0.0067) were also found in confirmed cases. There was no statistical difference among anorexia, heartburn, and hematochezia because they are rarely observed (*p* > 0.05).

Moreover, we further surveyed detailed characteristics for diarrhea and vomit symptoms. As Figure 2(b) has shown, no difference was found in stool frequency (3.07 ± 0.24 vs. 2.67 ± 0.42 per day, *p* = 0.48). However, among those who presented with diarrhea, 56% confirmed patients described watery diarrhea while only 16.7% reported watery stool in excluded cases (*p* < 0.0001, Figure 2(c)). With regard to vomit symptom, confirmed COVID-19 patients also had higher frequent vomit than the excluded cases (2.77 ± 0.97 vs. 1.80 ± 0.45 per day, *p* = 0.041), and they reported higher rates of nonfood or drug-related percentage of disordered vomit (59.09% vs. 40%, *p* < 0.001) (Figure 2(e)).

## 4. Discussion

The study presents the clinical characteristics of inquiries about digestive symptoms by doctors in different specialties and found that gastroenterologists could detect a greater proportion of gastrointestinal symptoms in COVID-19 patients during fever clinic. Moreover, COVID-19 patients are prone to present higher severity in digestive symptoms.

Gastroenterologists inquired a greater proportion of digestive symptoms in COVID-19 patients. This might be accounted by several reasons. Firstly, typical clinical symptoms of virus infection developed with fever and cough. Severe acute respiratory infection symptoms could be observed, with some patients rapidly developing acute respiratory distress syndrome (ARDS) and acute respiratory failure [12]. Therefore, the emphasis of the physician's inquiry was focused on respiratory symptoms. Secondly, in the early stage of the disease, none of us had much experience and gastrointestinal symptoms were not frequently observed in patients with COVID-19. It thereby may be easily misdiagnosed and ignored, especially for patients with diarrhea and other gastrointestinal symptoms as the initial appearance [7]. Thirdly, doctors with nondigestive specialists may further neglect digestive system inquiries. Our recent work demonstrated that these patients have a longer delay before viral clearance and to experience delayed diagnosis compared to patients with respiratory symptoms. More importantly, patients with digestive symptoms were more likely to have severe disease and worse prognosis than patients that did not have digestive symptoms [13]. Therefore, the digestive involvement of 2019-nCoV should be recognized in detail in outpatients.

Our study further found that in COVID-19 patients who presented with similar respiratory symptoms inquired by gastroenterologists, the confirmed cases were more likely to have digestive symptoms, such as diarrhea, vomiting, distention, and abdominal pain than the excluded ones. It has been found that angiotensin-converting enzyme II (ACE2) is the receptor of 2019-nCoV into cells and is highly expressed in the small intestine, duodenum, and colon [14, 15], suggesting that the digestive tract could be the target organ of 2019-nCoV. The SARS-CoV-2 directly damage the gut mucosa, leading to gastrointestinal symptoms. Our study also suggested that digestive system involvement could be another main symptom of COVID-19.

In addition, our study indicated that only nearly 40% of patients in the fever clinic were diagnosed with COVID-19 while the rest were other common diseases such as influenza, pharyngitis, common cold, or bacterial pneumonia. This result suggested that other respiratory epidemic cases might be regarded as COVID-19 and sent to fever clinic, arising risk for cross-infection.

Our study has some limitations. First, due to lack of nucleic test kit in the early epidemic, clinical diagnosis by CT examination was used as a high-sensitive and quick early diagnose, so false-positive results may be produced. Secondly, it would have been better to include as many patients as possible in Wuhan, in other cities in China, and even in other countries in order to get a more comprehensive understanding of outpatients with the digestive system. However, the data in this study permitted an early outcome assessment of the clinical characteristics of gastrointestinal findings with COVID-19 in the fever clinic and can be valuable for those who need early triage through inquiry. Finally, the study did not explore psychiatric comorbidity that was reported in COVID-19 patients which is discussed by Hao et al. and psychiatric comorbidity indeed could also induce multiple digestive symptoms [16]. Although gastroenterologists may pay less attention to COVID-19 symptoms of other systems (respiratory, cardiac, etc.), physicians in the fever clinic paying more attention to the inquiries of gastrointestinal symptoms in suspected COVID-19 patients is the main purpose of our article. Of course, if conditions permit, initial inquiry of COVID-19 cases requires participation of various specialists.

## 5. Conclusions

In conclusion, we found that gastroenterologists could reveal more proportion of gastrointestinal symptoms in COVID-19 patients during fever clinic inquiries. Physicians in a fever clinic should pay more attention to the inquiries of gastrointestinal symptoms in suspected COVID-19 patients.

## Figures and Tables

**Table 1:** Baseline characteristics of included COVID-19 patients based on the classification of different outpatient doctors.

	Total (*N* = 1238)	Reception by gastroenterologist (*N* = 611)	Reception by nongastroenterologist (*N* = 627)	*p*
Age (years)	42.24 ± 16.99	42.85 ± 17.31	41.10 ± 16.81	0.07
Gender				0.39
Male	515	261	254	
Female	723	350	373	
Fever				
Temperature (°C)	38.33 ± 0.95	38.31 ± 1.60	38.44 ± 0.99	0.17
Comorbidity				
Hypertension	53	27 (4.42%)	26 (4.15%)	0.71
Diabetes	23	12 (1.96%)	11 (1.75%)	0.77
Cerebrovascular disease	26	13 (2.13%)	13 (2.07%)	0.93
Digestive disease	6	4 (0.65%)	2 (0.32%)	0.39
Others^∗^	43	24 (3.93%)	19 (3.03%)	0.37
Before outpatient (days)		2.25 ± 1.29	2.12 ± 1.95	0.19
Initial symptoms				
Fever	958	463 (75.78%)	495 (78.95%)	0.18
Cough	141	70 (11.46%)	71 (11.32%)	0.94
Chest pain	9	4 (0.65%)	5 (0.78%)	0.77
Shortness of breath	21	14 (2.29%)	7 (1.11%)	0.11
Fatigue	24	14 (2.29%)	10 (1.59%)	0.37
Muscle soreness	12	7 (1.45%)	5 (0.80%)	0.53
Diarrhea	7	5 (0.82%)	2 (0.32%)	0.24
Others^#^	66	34 (5.64%)	32 (5.10%)	0.72
Confirmed COVID-19 cases	495	254 (41.57%)	241 (38.43%)	0.27

Others^∗^ included neurological, hematological, nephrological, and dermatological diseases. Others^#^ included dizziness, headache, and rhinorrhea. *p* values were compared between the two groups. A significance level of *p* ≤ 0.05 was used.

**Table 2:** To compare the incidence of digestive symptoms inquired between gastroenterologists and nongastroenterologists.

Items	Total (*N* = 495)	Reception by gastroenterologist (*N* = 254)	Reception by nongastroenterologist (*N* = 241)	*p*
Total digestive symptoms	76	58 (22.83%)	18 (7.47%)	<0.0001
Diarrhea	29	25 (9.84%)	4 (1.66%)	0.0001
Abdominal pain	10	7 (2.76%)	3 (1.24%)	0.23
Distention	15	12 (4.72%)	3 (1.24%)	0.02
Heartburn	1	1 (0.39%)	0 (0%)	0.32
Vomit	35	22 (8.66%)	13 (5.39%)	0.16
Anorexia	7	6 (0.23%)	1 (0.41%)	0.06
Hematochezia	1	1 (0.39%)	0 (0%)	0.32

Note: *p* values were compared between the two groups. A significance level of *p* ≤ 0.05 was used.

## Data Availability

The data used to support the findings of this study are included within the article and are also available on request through houxh@hust.edu.cn.
